# Soluble Urokinase-Type Plasminogen Activator Receptor Plasma Concentration May Predict Susceptibility to High Altitude Pulmonary Edema

**DOI:** 10.1155/2016/1942460

**Published:** 2016-06-09

**Authors:** Matthias Peter Hilty, Stefanie Zügel, Michele Schoeb, Katja Auinger, Christoph Dehnert, Marco Maggiorini

**Affiliations:** ^1^Medical Intensive Care Unit, University Hospital of Zurich, 8091 Zurich, Switzerland; ^2^Institute for Sports Medicine, Ruprecht-Karls University of Heidelberg, 69117 Heidelberg, Germany

## Abstract

*Introduction.* Acute exposure to high altitude induces inflammation. However, the relationship between inflammation and high altitude related illness such as high altitude pulmonary edema (HAPE) and acute mountain sickness (AMS) is poorly understood. We tested if soluble urokinase-type plasminogen activator receptor (suPAR) plasma concentration, a prognostic factor for cardiovascular disease and marker for low grade activation of leukocytes, will predict susceptibility to HAPE and AMS.* Methods.* 41 healthy mountaineers were examined at sea level (SL, 446 m) and 24 h after rapid ascent to 4559 m (HA). 24/41 subjects had a history of HAPE and were thus considered HAPE-susceptible (HAPE-s). Out of the latter, 10/24 HAPE-s subjects were randomly chosen to suppress the inflammatory cascade with dexamethasone 8 mg bid 24 h prior to ascent.* Results.* Acute hypoxic exposure led to an acute inflammatory reaction represented by an increase in suPAR (1.9 ± 0.4 at SL versus 2.3 ± 0.5 at HA, *p* < 0.01), CRP (0.7 ± 0.5 at SL versus 3.6 ± 4.6 at HA, *p* < 0.01), and IL-6 (0.8 ± 0.4 at SL versus 3.3 ± 4.9 at HA, *p* < 0.01) in all subjects except those receiving dexamethasone. The ascent associated decrease in PaO_2_ correlated with the increase in IL-6 (*r* = 0.46, *p* < 0.001), but not suPAR (*r* = 0.27, *p* = 0.08); the increase in IL-6 was not correlated with suPAR (*r* = 0.16, *p* = 0.24). Baseline suPAR plasma concentration was higher in the HAPE-s group (2.0 ± 0.4 versus 1.8 ± 0.4, *p* = 0.04); no difference was found for CRP and IL-6 and for subjects developing AMS.* Conclusion.* High altitude exposure leads to an increase in suPAR plasma concentration, with the missing correlation between suPAR and IL-6 suggesting a cytokine independent, leukocyte mediated mechanism of low grade inflammation. The correlation between IL-6 and PaO_2_ suggests a direct effect of hypoxia, which is not the case for suPAR. However, suPAR plasma concentration measured before hypoxic exposure may predict HAPE susceptibility.

## 1. Introduction

High altitude related diseases such as high altitude pulmonary edema (HAPE) and acute mountain sickness (AMS) play an important role for an increasing number of mountaineers and workforce operating at high altitude. Preventive efforts include limiting the rate of ascent to 300–600 m/day [1, 2] and administering medication before ascent such as nifedipine or tadalafil to prevent HAPE [3, 4] or acetazolamide to prevent AMS [5] in persons at risk. However, identification of risk factors has been proven difficult. Measurements taken during hypoxic exposure such as low hypoxic ventilatory response [6] and accentuated hypoxic pulmonary vasoconstriction [7] are only loosely associated with HAPE risk. No relation has been found to baseline factors such as age [8], gender [9] or genetic predisposition [10, 11] and only loose association of small lung volume and body mass index in the case of HAPE [12] or AMS [9]. Thus, HAPE susceptibility (HAPE-s) is currently considered in the presence of previous occurrence based on a recurrence rate of around 60% per further rapid ascent [13]. To a lesser degree the same holds true for AMS [14].

Inflammatory response to acute hypoxic stimulus, represented by increased blood concentration of eicosanoids [15], c-reactive protein (CRP), and cytokines such as tumor necrosis factor a (TNF-a) and interleukins 1, 2, and 6 (IL-1, IL-2, and IL-6) [16, 17], as well as the urinary concentration of eicosanoids [18], seems to play a role in the pathophysiology of high altitude illness. Higher levels of these inflammatory markers are seen in subjects developing AMS as compared to healthy controls at high altitude although no direct correlation between inflammatory markers and the magnitude of AMS has been established so far [16, 18]. While mechanical damage to the alveolar capillaries has been identified as the primary etiologic mechanism of the development and progression of HAPE, a secondary local inflammatory response is triggered [19]. Furthermore, preexisting subclinical inflammation during viral upper airway infection is associated with acquired HAPE susceptibility in children [20, 21] and in rats [22]. The former and the latter demonstrate a modulation of the mechanisms underlying HAPE by inflammatory processes.

With soluble urokinase-type plasminogen activator receptor a novel biomarker has been identified which provides a cellular centric view on the activation of the inflammatory cascade. suPAR is the cleaved and bloodborne form of the glycosylated membrane protein urokinase-type plasminogen activator receptor (uPAR) which is expressed on the cellular membrane mainly of leukocytes [23, 24]. The mechanisms triggering the expression of uPAR in different cell types and the cleavage of the membrane-bound protein to form soluble uPAR (suPAR) are still largely unknown [23]. suPAR plasma concentration has been found to serve as a diagnostic, prognostic, and treatment response parameter in various infectious and inflammatory diseases including sepsis [25] as well as a marker for chronic low grade inflammation. In the latter setting suPAR has been identified as a reliable predictor of cardiovascular risk in a general population [26, 27].

Given the involvement of inflammation in HAPE and AMS the aim of the present study is to test the hypothesis whether exposure to acute hypobaric hypoxia leads to an increase in suPAR plasma concentration comparable to that in CRP and IL-6 and whether suPAR as a marker of low level leukocyte activation predicts susceptibility to HAPE or AMS. To test our hypothesis, blood samples obtained in a previous study [28] from healthy mountaineers during rapid ascent to 4559 m were analyzed. suPAR plasma concentration at baseline and its development were then compared to the susceptibility to HAPE as determined by the subjects' history of exposure to high altitude and to the incidence of AMS during hypoxic exposure during the course of the study.

## 2. Methods

The data and the blood samples analyzed in this study originate from a research project evaluating early and late dexamethasone administration as HAPE prophylaxis. Three groups of mountaineers received either dexamethasone 8 mg bidaily starting twenty-four hours before or after ascent or no dexamethasone at all. Subjects were all confined to the same altitude profile and ascended within 24 hours to 4559 m and remained at altitude for four days. Thus in the twenty subjects having received dexamethasone prophylaxis starting after the first 24 hours at altitude further development of HAPE incidence, clinical presentation and biomarkers after the first 24 hours at altitude were influenced by the intervention. In order to avoid these confounders, the observation period at altitude in the present study is limited to 24 hours. The ten subjects having received dexamethasone prophylaxis before ascent serve to describe the effects of dexamethasone prophylaxis in this study. The study has been approved by the ethics committee of the University of Zurich (EK-1677) and was conducted in accordance with the declaration of Helsinki.

### 2.1. Study Population and Design

**Figure 1:**
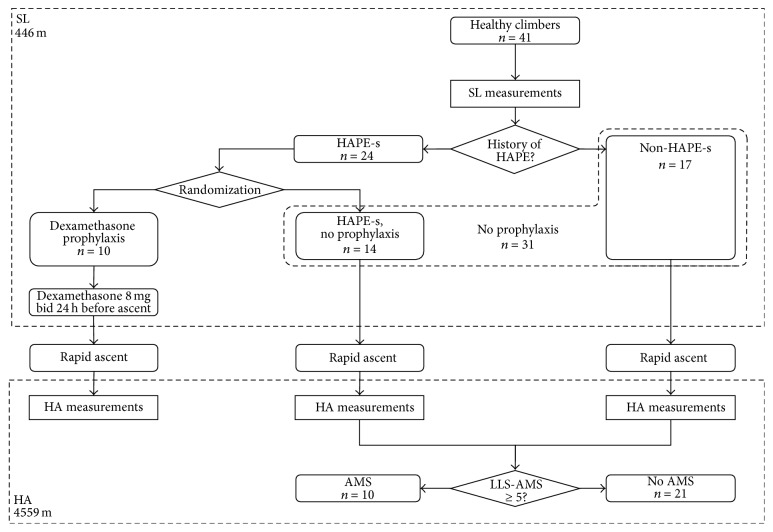
Study protocol. SL: sea level, HA: high altitude, HAPE: high altitude pulmonary edema, HAPE-s: high altitude pulmonary edema susceptibility, AMS: acute mountain sickness, and bid: two times per day.

Forty-one healthy mountaineers with climbing experience in the Alps (aged 44.8 ± 9.0 years, weight 72.7 ± 10.3 kg, height 174 ± 8 cm, BMI 24.1 ± 2.8 kg/m^2^, 29 (71%) males) were included in this study. Twenty-four of all subjects were HAPE-susceptible based on previous occurrence and were included in the “HAPE-s” group (*n* = 24), while the remaining seventeen subjects without history of HAPE were included in the “Non-HAPE-s” group (*n* = 17). Ten subjects belonging to the “HAPE-s” group were randomly selected to receive oral administration of dexamethasone 8 mg bidaily beginning twenty-four hours before ascent to form the “dexamethasone prophylaxis” group (*n* = 10), enabling comparison to the remaining subjects forming the “No prophylaxis” group (*n* = 31) (Figure 1). There were no differences in age, BMI, or gender distribution between either of the groups. All subjects underwent clinical examination and arterial blood sampling at the University of Zurich (sea level, SL; 446 m). Subjects were required to have spent less than three nights above 2500 m within a month preceding SL examinations in order to preclude acclimatization at the time of inclusion. Thereafter, within one week, all subjects ascended by foot in <24 hours to a mountain hut located on Signalkuppe peak on the Swiss-Italian border, the Margherita hut (high altitude, HA; 4559 m). They were observed at altitude for 24 hours.

### 2.2. Clinical and Laboratory Examination

Blood sampling as well as assessment for HAPE, including chest X-ray and clinical examination, and for AMS was performed at SL and repeated on the morning after arrival at HA. The occurrence of AMS was assessed using the Lake Louise Score for acute mountain sickness (LLS-AMS) with a cutoff value for the presence of AMS being defined as LLS-AMS ≥ 5 [29, 30]. Arterial blood was sampled for on-site analysis of blood gases (ABL800 Flex, Radiometer, Copenhagen, Denmark), particularly oxygen saturated hemoglobin fraction (SaO_2_) and oxygen partial pressure (PaO_2_), and for immediate centrifugation and freezing at −80°C. CRP, IL-6, and suPAR plasma concentration were then determined from the frozen samples, the latter using the suPARnostic*™* double monoclonal antibody sandwich enzyme-linked immunosorbent assay (Virogates, Copenhagen, Denmark). ELISA measurements were performed in duplicate; the mean of both measurements is reported.

### 2.3. Statistical Analysis

Comparison of population characteristics and inflammatory markers at SL versus HA was performed using the Wilcoxon signed rank test. Receiver operating characteristics analysis and a comparison between properties of “HAPE-s” and “Non-HAPE-s” groups at baseline by Mann-Whitney *U* test were used for assessment of the inflammatory markers' predictive properties for development of high altitude illness. Comparisons between “AMS” and “No AMS” groups at HA and retrospected to SL were also performed using the Mann-Whitney *U* test. Categorical population attributes were compared using Fisher's exact test. For correlation of inflammatory marker concentrations Pearson's product-moment correlation coefficient was used. A two-sided *p* < 0.05 was considered statistically significant. For all statistical analysis a fully scripted data management pathway was created within the R environment for statistical computing, version 2.15.2 [31]. Receiver operating characteristics analysis was performed using the R library ROCR version 1.0.5 [32]; graphical output was generated using the R library ggplot2, version 0.9.3.1 [33]. Values are given as mean ± SD.

## 3. Results

### 3.1. Measurements at Sea Level and Acute Exposure to Hypobaric Hypoxia

All subjects had similar values for arterial blood oxygenation, heart rate, LLS-AMS, suPAR, CRP, and IL-6 at SL (Table 1). In subjects not receiving dexamethasone prophylaxis acute exposure to hypobaric hypoxia led to a decrease in SaO_2_ and PaO_2_, as well as an increase in heart rate, LLS-AMS, suPAR, CRP, and IL-6 from SL to twenty-four hours after arrival at HA (Table 1). As the most direct marker for hypoxia the ascent associated decrease in PaO_2_ correlated with the increase in IL-6 (*r* = 0.46, *p* < 0.001) and CRP (*r* = 0.43, *p* < 0.001) but not suPAR (*r* = 0.27, *p* = 0.08). Furthermore, the increase in IL-6 correlated with the increase in CRP (*r* = 0.63, *p* < 0.001), but not suPAR with IL-6 (*r* = 0.16, *p* = 0.24) or CRP (*r* = 0.12, *p* = 0.37). In the “dexamethasone prophylaxis” group the decrease in SaO_2_ and PaO_2_ was attenuated but still present, while the high altitude mediated increase in heart rate, suPAR, and CRP was altogether inhibited. The increase in total LLS-AMS remained unchanged while the increase in the self-assessment subscore for headache (SA-HA) and global functionality (SA-FN), as well as all clinical assessment subscores, was inhibited (Table 1).

### 3.2. Prediction of HAPE Susceptibility

**Figure 2:**
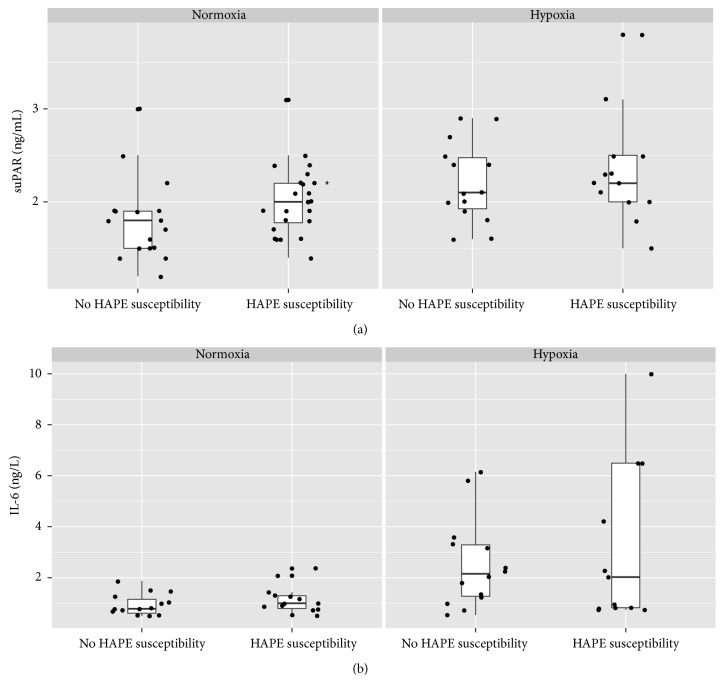
suPAR (a) and IL-6 (b) plasma concentrations in normoxia and hypoxia, in HAPE-susceptible versus non-HAPE-susceptible persons. (*∗*) denotes a difference in HAPE versus HAPE-susceptible subjects (*p* < 0.05). Boxplots represent median, interquartile range, and range. Horizontal scattering is applied to the individual data points in order to avoid superimposition. HAPE: high altitude pulmonary edema, suPAR: soluble urokinase-type plasminogen activator receptor, and IL-6: interleukin 6.

**Figure 3:**
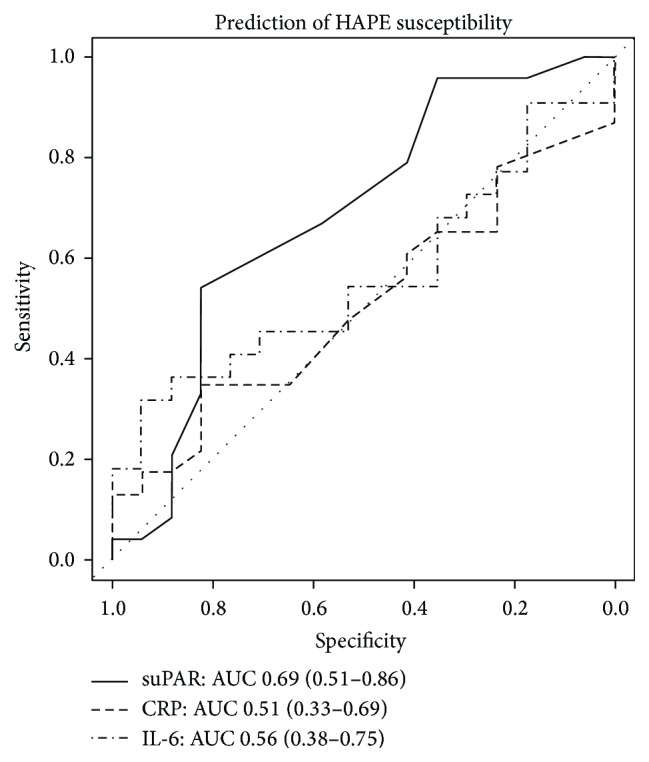
Receiver operating characteristics analysis for prediction of high altitude illness versus control group assignment by baseline measurements. AUC: area under the curve; in parentheses: 95% confidence interval.

At SL, subjects in the “HAPE-susceptible” and “non-HAPE-susceptible” groups presented with the same heart rate, PaO_2_, and LLS-AMS as well as CRP and IL-6 plasma concentrations (Table 2). There were no differences in age, BMI, or gender distribution between the two groups. However, HAPE-susceptible subjects had a higher baseline suPAR plasma concentration, as well as a higher SaO_2_ (Figure 2). Receiver operating characteristics analysis for the prediction of HAPE susceptibility yields an area under the curve of 0.69 for suPAR, 0.51 for CRP, and 0.56 for IL-6 (Figure 3). At HA, however, there was no difference in suPAR as well as any of the other parameters between the “HAPE-susceptible” and “non-HAPE-susceptible” groups. In the 24-hour observation period at altitude, no HAPE was detected.

### 3.3. Incidence of AMS and Prediction of AMS Susceptibility

Based on the presence of AMS at HA all subjects that have not received dexamethasone (all subjects in the “No prophylaxis” group) were reassigned either to the “AMS” group (*n* = 10) or the “No AMS” group (*n* = 21). There were no differences in age, BMI, or gender distribution between the two groups. 9/10 subjects (90%) having developed AMS at HA belonged to the Non-HAPE-s group. LLS-AMS at HA was approximately twice as severe in subjects assigned to the “AMS” group due to manifested AMS as compared to the control group (Table 3). However, there was no difference in any of the other parameters such as heart rate, SaO_2_, and PaO_2_, as well as suPAR, CRP, and IL-6 plasma concentrations. Also in retrospective analysis of the examination at SL there was no differentiation between the “AMS” group and the “No AMS” group in heart rate, SaO_2_, PaO_2,_ and LLS-AMS, as well as suPAR, CRP, and IL-6 plasma concentrations (Table 3). The overall LLS-AMS score increased regardless of dexamethasone prophylaxis from SL to HA by about four points (Table 1); the incidence of LLS-AMS ≥ 5 was 10/31 (32%) in the “No prophylaxis” group and 1/10 (10%) in the “dexamethasone prophylaxis” group (*p* = 0.33). The high altitude mediated increase in the LLS-AMS self-assessment subscore for headache (SA-HA) and global functionality (SA-FN), as well as all clinical assessment subscores, was inhibited by dexamethasone prophylaxis.

## 4. Discussion

The novel finding in the present study is that elevated suPAR at baseline may be a marker of HAPE susceptibility by differentiating HAPE and HAPE-s populations but does not identify subjects developing AMS during hypoxic exposure. Furthermore, the hypoxia mediated increase in suPAR is not related to acute inflammatory markers such as IL-6 and CRP.

Our study confirms the previously described [16, 17] increase in CRP and IL-6 plasma concentrations during acute hypoxic exposure and shows a correlation between the two. It further demonstrates an increase in suPAR plasma concentration; however it neither correlated to the increase in CRP nor correlated to the increase in IL-6. A possible explanation for this could be that the mechanism leading to cleavage of suPAR is different from generally understood processes leading to inflammation as reflected by CRP and IL-6. This is supported by the fact that suPAR cleavage is closely related to leukocyte migration as opposed to the on-demand synthesis of other inflammatory markers [23]. Furthermore, while the hypoxia-induced increase in CRP and IL-6 plasma concentrations correlated with the decrease in PaO_2_, this was not the case for suPAR, suggesting a different trigger for suPAR cleavage than a direct effect of hypoxia. Both the hypoxia-induced increase in CRP and the hypoxia-induced increase in suPAR were inhibited by dexamethasone prophylaxis along with the development of headache as the main symptom of AMS, as well as global functionality and the clinical assessment of mental status, ataxia, and peripheral edema, in concordance with previous data [34]. The lack of a statistically significant effect of dexamethasone prophylaxis on total LLS-AMS stems from the similar self-assessment of fatigue and dizziness between subjects with and without dexamethasone prophylaxis, as well as the only implied effect of the prophylaxis on the self-assessment of gastrointestinal symptoms and quality of sleep. The latter may be due to the relatively small number of subjects in the “dexamethasone prophylaxis” group and the possible bias introduced by a self-assessment score in general.

The difference in suPAR plasma concentration at sea level found in this study supports that HAPE susceptibles are a group of persons with a low grade inflammation that may encourage the development of HAPE once exposed to the stimulus of hypoxia. It is thus implied that HAPE susceptibility may be predicted without previous high altitude exposure by determining suPAR plasma concentration as a measure of such low grade inflammation, though with a limited sensitivity and specificity. This finding further suggests that low grade leukocyte activation enhances lung capillary vulnerability to hydrostatically induced leakage [19]. Such a low grade inflammatory condition may be a component of HAPE susceptibility, which is in agreement with observations of higher HAPE incidence related to preexisting inflammatory conditions [20–22]. Thus, the mechanisms underlying HAPE seem to be modulated, rather than caused, by inflammatory processes. At high altitude such a difference is not discernible anymore, possibly being inundated by the acute hypoxic stimulus. Despite an increase during hypoxic exposure suPAR plasma concentrations before ascent were not different between the groups later developing AMS or not. This suggests that cellular based inflammation does not play a role on the central form of high altitude disease, comprising AMS and high altitude cerebral edema.

Limitations of our study include being a field study and noncontrollable changes in the environment at the Capanna Margherita as opposed to the valley, such as temperature or sun exposure. In the present study we have minimized these influences by implementing a fast ascent profile and a strictly controlled daily routine within the protected environment of the Capanna and by obtaining HA measurements after a full night's rest following ascent. However, the observation period of 24 hours at altitude may be insufficient to exclude a further rise in inflammatory markers, especially CRP. Since HAPE incidence could only be assessed in the first 24 hours after hypoxic exposure as well, the analysis in the present study was mainly based on previous history. Furthermore, previous data suggests that in a general population suPAR plasma concentration seems to be higher in women than in men [26]. It is unlikely however that this is of relevance for our data since in the present study gender distribution is unrelated to HAPE susceptibility. The evaluation of the presence of AMS on the other hand, being based on a combination of self-assessment and clinical assessment is in theory susceptible to bias in self-assessment. A study in a larger population or at greater altitude is needed to confirm the predictive value of baseline suPAR measurements for HAPE susceptibility and reassess it in the case of susceptibility to AMS.

In conclusion, high altitude exposure leads to an increase in suPAR plasma concentration, with the missing correlation between suPAR and IL-6 suggesting a cytokine independent, leukocyte mediated mechanism of low grade inflammation. The correlation between IL-6 and PaO_2_ suggests a direct effect of hypoxia, which is not the case for suPAR. However, suPAR plasma concentration measured before hypoxic exposure may predict HAPE susceptibility, suggesting the presence of low grade inflammation as a predisposing factor for HAPE.

## Figures and Tables

**Table 1:** The effect of acute hypoxic exposure on inflammatory markers and physiological parameters with and without dexamethasone prophylaxis.

	No high altitude illness prophylaxis		*p*	Dexamethasone prophylaxis		*p*	Δ (hypoxia − normoxia)		*p*
	Normoxia	Hypoxia		Normoxia	Hypoxia		No prophylaxis	Dexamethasone prophylaxis	
	*n* = 31	*n* = 31		*n* = 10	*n* = 10		*n* = 31	*n* = 10	
Heart rate (1/min)	66 ± 10	82 ± 12	<0.001	74 ± 14	68 ± 16	0.65	16 ± 12	−7 ± 21	<0.01
SaO_2_ (%)	96 ± 1	76 ± 6	<0.001	96 ± 1	87 ± 3	<0.01	−20 ± 6	−9 ± 3	<0.001
PaO_2_ (kPa)	12.2 ± 1.6	5.3 ± 0.6	<0.001	11.4 ± 1.4	6.5 ± 0.7	<0.01	−7.1 ± 1.3	−4.9 ± 1.1	<0.001
LLS-AMS (1)	1 ± 1	5 ± 3	<0.001	1 ± 1	4 ± 2	<0.01	4 ± 3	3 ± 2	0.13
(i) SA-HA	0 ± 0	1 ± 1	<0.001	0 ± 0	0 ± 1	0.48	1 ± 1	0 ± 1	0.03
(ii) SA-GI	0 ± 0	0 ± 1	0.001	0 ± 0	0 ± 0	1	0 ± 1	0 ± 0	0.07
(iii) SA-FT	0 ± 0	1 ± 1	<0.001	0 ± 0	1 ± 1	0.13	1 ± 1	0 ± 1	0.14
(iv) SA-DZ	0 ± 0	1 ± 1	<0.001	0 ± 0	0 ± 0	0.35	1 ± 1	0 ± 0	0.11
(v) SA-SL	1 ± 1	2 ± 1	<0.001	0 ± 1	2 ± 1	0.005	1 ± 1	2 ± 1	0.08
(vi) SA-FN	0 ± 0	1 ± 1	<0.001	0 ± 0	0 ± 0	0.15	1 ± 1	0 ± 0	0.03
(vii) CA-MS	0 ± 0	0 ± 0	<0.001	0 ± 0	0 ± 0	0.65	0 ± 0	0 ± 0	0.002
(viii) CA-AT	0 ± 0	0 ± 0	<0.001	0 ± 0	0 ± 0	0.65	0 ± 0	0 ± 0	0.002
(ix) CA-ED	0 ± 0	0 ± 1	<0.001	0 ± 0	0 ± 0	0.65	0 ± 1	0 ± 0	0.002
suPAR (ng/mL)	1.9 ± 0.4	2.3 ± 0.5	<0.001	2.0 ± 0.3	2.0 ± 0.3	0.61	0.3 ± 0.3	0.0 ± 0.3	0.001
CRP (mg/L)	0.7 ± 0.5	3.6 ± 4.6	<0.001	2.4 ± 3.1	1.9 ± 2.6	0.21	3.0 ± 4.5	−0.5 ± 1.1	<0.001
IL-6 (ng/L)	0.8 ± 0.4	3.3 ± 4.9	<0.001	1.0 ± 0.7	0.6 ± 0.6	0.32	2.7 ± 5.0	−0.4 ± 1.1	<0.001

Values are given as mean ± SD. SaO_2_: oxygen saturated hemoglobin fraction and LLS-AMS: Lake Louise score for acute mountain sickness; the cutoff value for the presence of AMS is defined as LLS-AMS ≥ 5 [29, 30]; LLS-AMS self-assessment subscores (scale from 0 to 3): SA-HA: self-assessment headache subscore, SA-GI: self-assessment gastrointestinal subscore, SA-FT: self-assessment fatigue subscore, SA-DZ: self-assessment dizziness subscore, SA-SL: self-assessment sleep quality subscore, and SA-FN: self-assessment global functionality subscore; LLS-AMS clinical assessment subscores: CA-MS: clinical assessment mental status subscore (scale from 0 to 4), CA-AT: clinical assessment ataxia subscore (scale from 0 to 4), and CA-ED: clinical assessment edema subscore (scale from 0 to 2).

**Table 2:** Inflammatory markers and physiologic parameters of HAPE-susceptible and non-HAPE-susceptible persons at sea level (normoxia) and during acute hypoxic exposure.

	Normoxia		*p*	Hypoxia		*p*
	Non-HAPE-s	HAPE-s		Non-HAPE-s	HAPE-s	
	*n* = 17	*n* = 24		*n* = 17	*n* = 14	
Heart rate (1/min)	64 ± 10	71 ± 12	0.07	79 ± 10	86 ± 13	0.13
SaO_2_ (%)	96 ± 1	97 ± 1	0.01	76 ± 4	76 ± 8	0.74
PaO_2_ (kPa)	11.8 ± 1.8	12.1 ± 1.4	0.81	5.3 ± 0.3	5.3 ± 0.8	1.00
LLS-AMS (1)	1 ± 1	1 ± 1	0.64	5 ± 3	5 ± 2	0.97
suPAR (ng/ml)	1.8 ± 0.4	2.0 ± 0.4	0.04	2.2 ± 0.4	2.3 ± 0.6	0.66
CRP (mg/L)	0.7 ± 0.5	1.4 ± 2.2	0.93	2.2 ± 1.7	5.0 ± 6.3	0.25
IL-6 (ng/L)	0.9 ± 0.4	0.9 ± 0.6	0.53	2.5 ± 1.7	4.1 ± 6.9	0.62

Values are given as mean ± SD. SaO_2_: oxygen saturated hemoglobin fraction and LLS-AMS: Lake Louise score for acute mountain sickness; the cutoff value for the presence of AMS is defined as LLS-AMS ≥ 5 [29, 30].

**Table 3:** Inflammatory markers and physiologic parameters of persons developing AMS versus those not developing AMS during acute hypoxic exposure and retrospectively at sea level (normoxia).

	Normoxia		*p*	Hypoxia		*p*
	No AMS	AMS		No AMS	AMS	
	*n* = 21	*n* = 10		*n* = 21	*n* = 10	
Heart rate (1/min)	65 ± 11	67 ± 10	0.75	83 ± 12	80 ± 11	0.47
SaO_2_ (%)	96 ± 1	96 ± 1	0.70	77 ± 5	73 ± 7	0.16
PaO_2_ (kPa)	12.4 ± 1.0	11.9 ± 2.4	0.73	5.4 ± 0.6	5.0 ± 0.5	0.12
LLS-AMS (1)	1 ± 1	2 ± 1	0.25	4 ± 2	8 ± 2	0.001
suPAR (ng/mL)	1.9 ± 0.5	2.0 ± 0.3	0.10	2.3 ± 0.6	2.3 ± 0.2	0.49
CRP (mg/L)	0.6 ± 0.5	0.8 ± 0.4	0.38	3.7 ± 5.3	3.4 ± 2.6	0.67
IL-6 (ng/L)	0.8 ± 0.5	0.8 ± 0.3	0.84	3.6 ± 5.8	2.6 ± 1.9	0.77

Values are given as mean ± SD. SaO_2_: oxygen saturated hemoglobin fraction and LLS-AMS: Lake Louise score for acute mountain sickness; the cutoff value for the presence of AMS is defined as LLS-AMS ≥ 5 [29, 30].
